# SATB1 establishes ameloblast cell polarity and regulates directional amelogenin secretion for enamel formation

**DOI:** 10.1186/s12915-019-0722-9

**Published:** 2019-12-13

**Authors:** Yan Zhang, Liwei Zheng, Michael Le, Yukiko Nakano, Barry Chan, Yulei Huang, Parisa Moravedje Torbaty, Yoshinori Kohwi, Ralph Marcucio, Stefan Habelitz, Pamela K. Den Besten, Terumi Kohwi-Shigematsu

**Affiliations:** 1 Department of Orofacial Sciences, University of California, San Francisco, USA; 2 Department of Orthopaedic Surgery, University of California, San Francisco, USA; 3 Preventive and Restorative Dental Sciences, University of California, San Francisco, USA

**Keywords:** Ameloblast, Enamel, Special AT-rich sequence-binding protein-1 (SATB1), Amelogenin, Claudin 1, Tight junction, Filamentous actin assembly, EPS8, Epithelial polarity, Membrane trafficking

## Abstract

**Background:**

Polarity is necessary for epithelial cells to perform distinct functions at their apical and basal surfaces. Oral epithelial cell-derived ameloblasts at secretory stage (SABs) synthesize large amounts of enamel matrix proteins (EMPs), largely amelogenins. EMPs are unidirectionally secreted into the enamel space through their apical cytoplasmic protrusions, or Tomes’ processes (TPs), to guide the enamel formation. Little is known about the transcriptional regulation underlying the establishment of cell polarity and unidirectional secretion of SABs.

**Results:**

The higher-order chromatin architecture of eukaryotic genome plays important roles in cell- and stage-specific transcriptional programming. A genome organizer, special AT-rich sequence-binding protein 1 (SATB1), was discovered to be significantly upregulated in ameloblasts compared to oral epithelial cells using a whole-transcript microarray analysis. The *Satb1*^*−/−*^ mice possessed deformed ameloblasts and a thin layer of hypomineralized and non-prismatic enamel. Remarkably, *Satb1*^*−/−*^ ameloblasts at the secretory stage lost many morphological characteristics found at the apical surface of wild-type (*wt)* SABs, including the loss of Tomes’ processes, defective inter-ameloblastic adhesion, and filamentous actin architecture. As expected, the secretory function of *Satb1*^−/−^ SABs was compromised as amelogenins were largely retained in cells. We found the expression of epidermal growth factor receptor pathway substrate 8 (*Eps8*), a known regulator for actin filament assembly and small intestinal epithelial cytoplasmic protrusion formation, to be SATB1 dependent. In contrast to *wt* SABs, EPS8 could not be detected at the apical surface of *Satb1*^*−/−*^ SABs. *Eps8* expression was greatly reduced in small intestinal epithelial cells in *Satb1*^−/−^ mice as well, displaying defective intestinal microvilli.

**Conclusions:**

Our data show that SATB1 is essential for establishing secretory ameloblast cell polarity and for EMP secretion. In line with the deformed apical architecture, amelogenin transport to the apical secretory front and secretion into enamel space were impeded in *Satb1*^*−/−*^ SABs resulting in a massive cytoplasmic accumulation of amelogenins and a thin layer of hypomineralized enamel. Our studies strongly suggest that SATB1-dependent *Eps8* expression plays a critical role in cytoplasmic protrusion formation in both SABs and in small intestines. This study demonstrates the role of SATB1 in the regulation of amelogenesis and the potential application of SATB1 in ameloblast/enamel regeneration.

## Background

The establishment of polarity is pivotal for many fundamental epithelial cellular functions. Epithelial polarity is characterized by cells with distinct apical and basolateral surface structural orientations and molecular localization patterns that allow cells to fulfill their specialized functions, for instance, small intestine epithelial cells uptake nutrients at their apical surface and attach to the basement membrane at the basal surface [1, 2].

Ameloblasts are specialized epithelial cells responsible for the formation of the enamel, the hardest tissue in the human body, acting to protect the teeth from wear, caries progression, and fracture. Ameloblast differentiation goes through a series of sequential morphological changes, which begins with the proliferating and thickening of the cuboidal oral epithelial cells lining the first branchial arches of the jaws at the presumptive tooth sites. In response to the signaling from the dental mesenchyme, cuboidal dental epithelial precursor cells differentiate into the epithelial lineage of the enamel organ, including a layer of inner enamel epithelial cells (IEEs), stratum intermedium, stellate reticulum, and a layer of outer enamel epithelial cells. IEEs progress to a single-cell layer of tightly packed and columnar presecretory ameloblasts (PABs) when the distal outer layer of the dental papilla differentiates into odontoblasts and secretes dentin matrix, in which the primary organic component is type I collagen [3]. The signaling cues from dental mesenchymal cells and dentin matrix facilitate further differentiation of PABs to morphologically and functionally distinct secretory ameloblasts (SABs).

SABs are a layer of tall columnar epithelial cells which have an average ratio of length to diameter of about 10. In these elongated cells, the nuclei shift toward the stratum intermedium at the basal surface, allowing the rough endoplasmic reticulum, Golgi complex, and secretory vesicles to occupy a large portion of the supranuclear compartment. Utilizing these organelles, SABs synthesize and package enamel matrix proteins (EMPs), composed of 90% amelogenins, into the vesicles and subsequently transport them to the apical surface of SABs. These vesicles fuse with the Tomes’ processes, which are six-sided pyramid-like cytoplasmic protrusion at the apical surface of SABs, and then EMPs are deposited into the extracellular enamel space [4, 5]. Cytoskeleton fibers play important roles in coordinating the directional movement of ameloblasts and their secretory vesicles [6]. Filamentous actin bundles shape Tomes’ processes, to form a secretory front to direct the polarized trafficking of secretory vesicles and orientation of enamel crystals, also referred to as enamel rods [7–11]. Enamel crystal rods are arranged in parallel arrays and form an intricate 3D pattern which creates flexibility and strength of the mineralized enamel matrix [12].

As the full thickness of the enamel matrix is deposited, secretory ameloblasts differentiate into maturation ameloblasts. These cells adopt a low columnar morphology and are primarily responsible for ion transport and reabsorption of water and peptides hydrolyzed from the enamel matrix proteins [13–15] to orchestrate the full mineralized enamel matrix that consists of 96% minerals. When enamel biomineralization is complete, ameloblasts subsequently either commit to apoptosis or become the junctional epithelial cells prior to tooth eruption [16, 17]. Hence, ameloblasts do not exist in adult human teeth.

To move toward our ultimate goal of regenerating an entire human tooth organ, it is essential to develop strategies to establish a reliable source of functional ameloblasts. Each developmental stage of ameloblasts has unique features in gene expression profile, morphology, cellular processes, and functions. Thus, the complexities of ameloblast differentiation pose a big hurdle to advance our goals for tooth regeneration. To better understand the stage-specific ameloblasts, first, we utilized the whole-transcript microarray analyses to identify the significantly differentially expressed transcriptional regulators in presecretory ameloblasts (PABs) and secretory ameloblasts (SABs), as compared to autologous oral epithelial cells (OEs) and human embryonic stem cell-derived epithelial cells (ES-ECs). Our studies showed that special AT-rich sequence-binding protein-1 (SATB1) [18] was upregulated in ameloblasts in a tissue- and stage-specific manner during tooth development. We focused our study on SATB1 because it has a global gene regulatory function as a genome organizer by regulating 3D chromatin architecture [19–26]. With the use of a *Satb1*^−/−^ mouse model [27], we demonstrate that indeed SATB1 is a critical regulator for ameloblast polarization, apical actin filament assembly, Tomes’ process formation, and enamel matrix protein secretion.

## Results

### Ameloblasts have cell type- and stage-specific gene expression profiles

Principal component analysis (PCA) of the normalized microarray data showed that the triplicate samples of each cell type (designated as ES-EC, OE, PAB, and SAB) were tightly distributed in a biplot, indicating the reproducibility of these assays. The reduced principal components of each cell type clustered together, and each had their own unique positions in the biplot (see Additional file 1: Figure S1), indicating that ameloblasts, as a type of highly specialized epithelial cells, did exhibit a distinctive gene expression pattern as compared to non-dental epithelial cells ES-EC and OE.

**Fig. 1:**
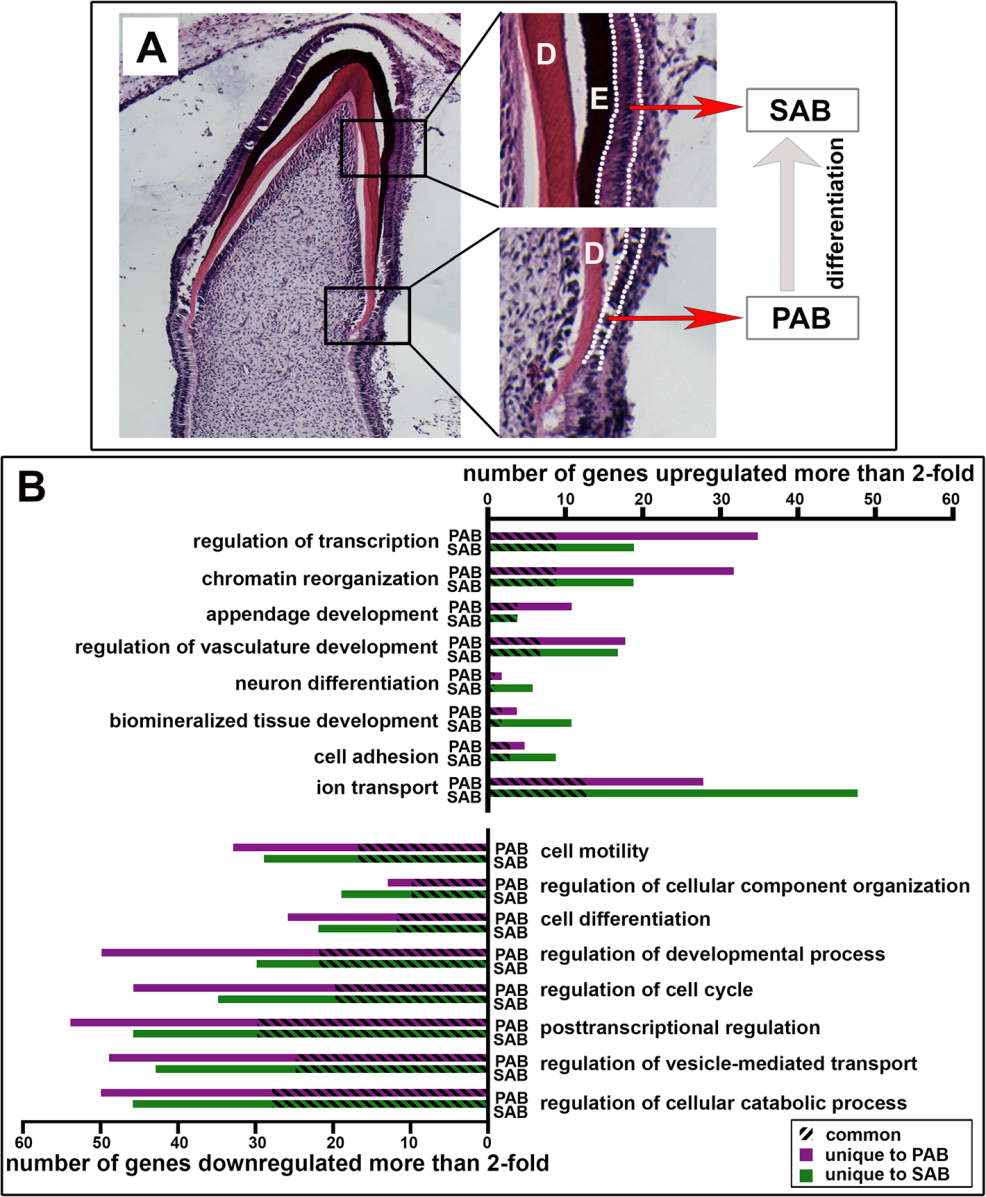
The unique expression patterns of presecretory ameloblasts and secretory ameloblasts are identified through a comparison whole-transcript expression microarray analysis. **a** The anatomic structure of presecretory ameloblasts (PAB) and secretory ameloblasts (SAB) that were collected from 18–19-week-old human developing incisors using laser capture microscope (LCM). Low columnar epithelial cells (between the dashed white lines in the lower right panel) next to the dentin matrix (D) were microdissected as PAB. Elongated and polarized epithelial cells (between the dashed white lines in the top right panel) adjacent to the organic enamel matrix (E) were microdissected as SAB. **b** The number of significantly upregulated (top) and downregulated (bottom) genes expressed by PABs and SABs as compared to non-dental epithelial cells in each of the top eight most significant GO terms, grouped by various Gene Ontology (GO) processes. A number of genes that were significantly altered in both PAB and SAB were labeled as common (identified by slashes), and the number of genes that were unique to either PAB or SAB was labeled in purple or in green

Differential gene expression analyses showed that there were 908 genes in PAB and 1203 genes in SAB that were either down- or upregulated more than 2.0-fold as compared to the average expression level of the same gene expressed by ES-EC and OE (*p* < 0.05). The most significantly upregulated genes in SAB or PAB were genes related to biomineralized tissue development, cell adhesion, ion transport, transcriptional regulation, epithelial appendage development, and neuron differentiation. It is not surprising that ameloblasts, epidermal cells, and neuronal cells share some degree of similarity, since they are all derived from the ectoderm. The most significantly downregulated genes were involved in cell motility, cellular component organization, cell cycle and regulation of cellular catabolic process, etc., indicating the terminal differentiation state of ameloblasts. The numbers of significantly up- or downregulated genes in several key gene ontology terms are illustrated in Fig. 1b. PAB and SAB shared many similar molecular phenotypes, as evidenced by the fact that many genes were commonly regulated in both cell types.

Next, we verified the expression levels of some ameloblast-associated marker genes by real-time PCR analysis (see Additional file 2: Figure S2), which confirmed the results from microarray analysis that enamel matrix proteins, including amelogenin, ameloblastin, and enamelin, were all significantly upregulated more than 50-fold in PAB and over 100-fold in SAB as compared to either ES-EC or OE. MMP-20, a specific enamel matrix proteinase [28], was upregulated 137-fold and 119-fold in SAB as compared to ES-EC and OE, respectively. MMP-20 increased about 6-fold in PAB as compared to either ES-EC or OE. Amelotin, an enamel matrix protein mostly produced by maturation ameloblasts [29], was also upregulated in SAB. Interestingly, dentin sialophosphoprotein (DSPP), alkaline phosphatase (ALP) ,and dentin matrix protein-1 (DMP1), which are also linked to dentin and bone formation, were all significantly upregulated in SAB as compared to ES-EC, OE, and PAB.

Microarray analysis showed that claudin 1 (CLDN1), a major component of tight junction complexes that can join epithelial cells together as a continuous sheet [30–32], was the most upregulated cell adhesion-associated gene in SAB. Further, qPCR analyses of microdissected cells confirmed that this gene was upregulated 23- and 30-fold in the PAB and SAB, respectively, as compared to ES-EC (see Additional file 2: Figure S2). These data imply that as compared to the flat and cuboidal ES-EC and oral epithelial cells, the super elongated columnar ameloblasts may require more sturdy tight junctions to maintain the rigidity and paracellular permeability of ameloblast layer. Integrin alpha 2 (ITGA2), previously identified as a major integrin mediating the attachment of early-stage ameloblasts to the basement membrane and dentin matrix [33], was also upregulated in PAB as compared to ES-EC, OE, and SAB based on both microarray and qPCR analyses.

The two most significantly upregulated transcription regulators identified through the microarray analysis, and confirmed by qPCR, were *SATB1* and *c-MAF*. It is worth mentioning that the microarray analysis also showed the significant upregulation of transcriptional regulator DLX3 in SABs. The functions of DLX3 in ameloblast differentiation and enamel formation have been previously identified [34–37].

### SATB1 distributes in the ameloblasts in a stage-specific manner

**Fig. 2:**
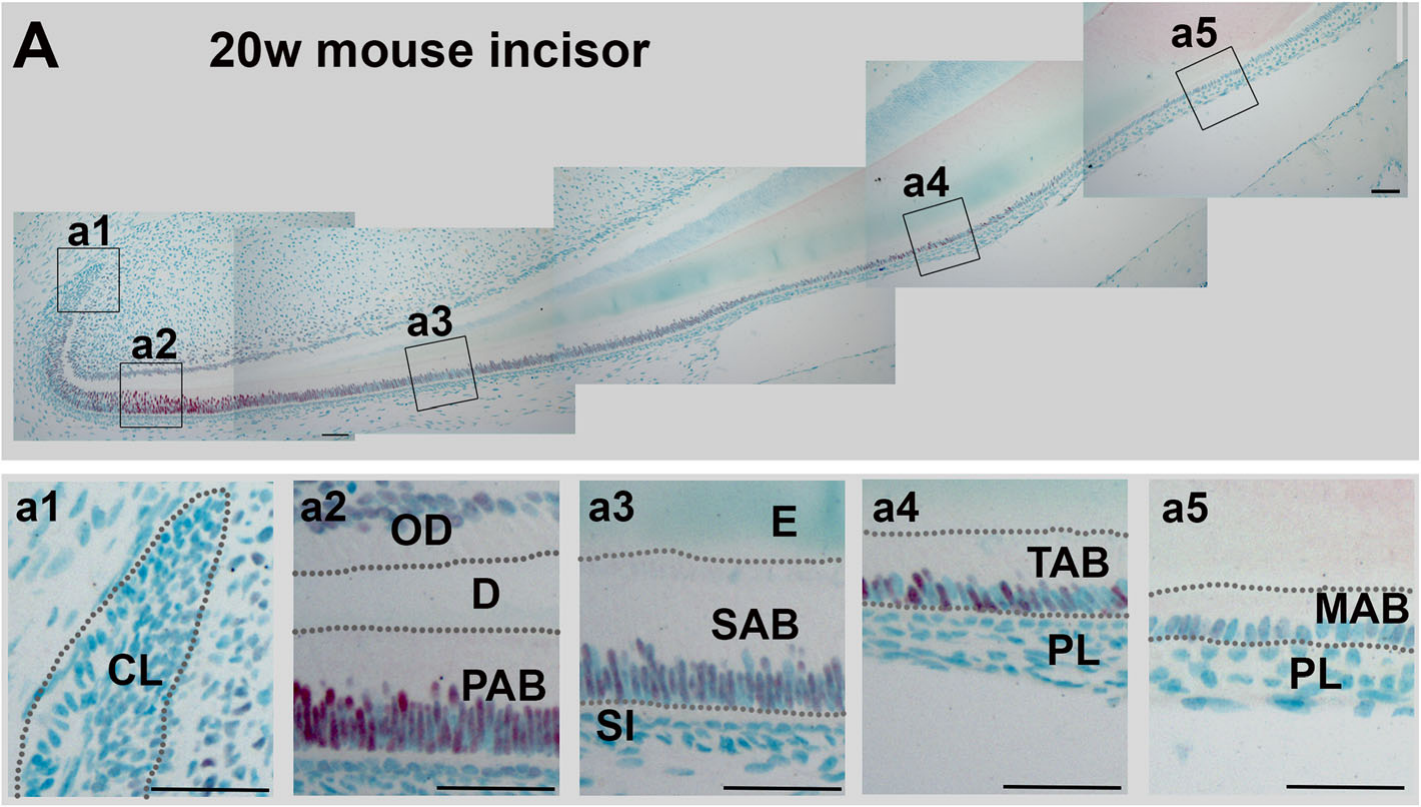
Immunohistochemical staining shows that SATB1 is present in mouse incisors in a cell- and stage-specific manner. In a representative sagittal section of the incisor from 20-week-old *wt* C57BL/6J female mouse, SATB1 immunostaining signal (in red) was intense in PAB (**a2**), reduced in SAB (**a3**), then moderately increased in transition stage ameloblasts (TAB) (**a4**), and significantly reduced last in maturation ameloblasts (MAB) (**a5**). SATB1 could not be detected in cells of the cervical loop (CL; **a1**), stratum intermedium (SI; **a3**), and papillary layer (PL; **a4** and **a5**). Scale bars in **a1**–**a5**, 50 μM

We chose to study the potential role of SATB1 in ameloblast/enamel formation because of its capacity to establish cell-specific transcription programs during the specific cell lineage development, including epidermal cells [38], T cells [21, 24, 26, 27, 39–43], postnatal brain [44], and pituitary gland [45]. SATB1 is also known to reprogram transcription profiles in tumor cells to promote tumor progression and metastasis [22, 46, 47]. Immunohistochemical staining showed that, during tooth development, SATB1 was only immunolocalized in the nuclei of ameloblast lineage cells, with the highest signal in PABs; relatively reduced in SABs; and moderately increased again in transition-stage ameloblasts, with a subsequent reduction in maturation ameloblasts (see Fig. 2).

### *Satb1* ablation results in a thin and hypomineralized enamel

**Fig. 3:**
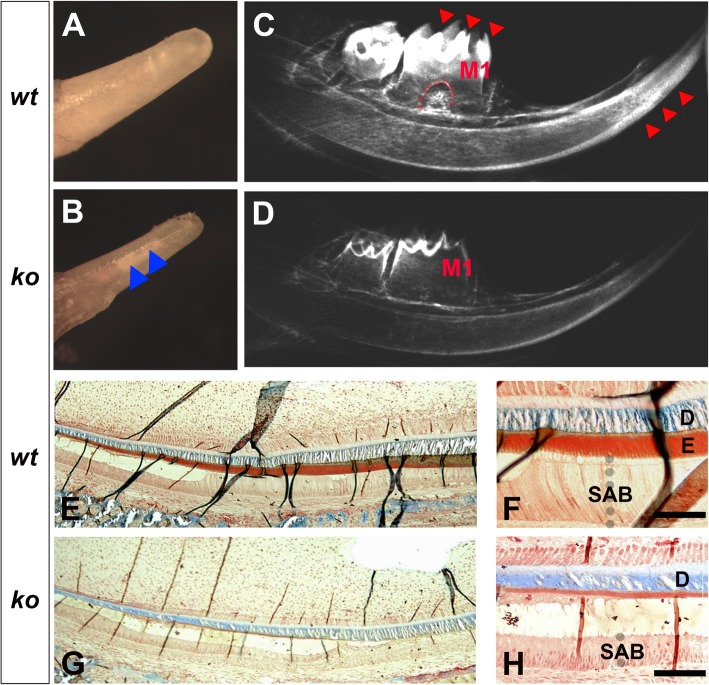
A thin and hypomineralized enamel layer is detected on the *Satb1*^*−/−*^ mouse teeth. The gross morphology shows that the erupted portion of the P13 *wt* mouse mandibular incisor (**a**) is larger than the diameter of the erupted portion of the P13 *Satb1*^*−/−*^ mouse mandibular incisor (**b**). The enamel layer of the *Satb1*^*−/−*^ mouse incisor is more transparent, allowing the underneath dentin to be seen (indicated by blue arrows in **b**). **c** X-ray radiographic image of one *wt* hemimandible shows that well-contrasted mineralized enamel (indicated by red triangles) is easily distinguishable from the dentin on the molars and incisor. In the *wt* mouse first molar (M1), the root furcation is visible, as marked by the dashed red line. **d** As to the *Satb1*^*−/−*^ mouse hemimandible, the X-ray radiographic image shows only the dentin to be seen; no furcation is detectable in M1. **e** Trichrome staining of the *wt* P13 incisor shows the mineralizing dentin layer stained in blue and enamel layer stained in red/brown. Wild-type secretory ameloblasts (SAB) (**f**), enamel (**e**), and dentin (**d**) are illustrated in the enlarged image. **g** In the P13 *Satb1*^*−/−*^ mouse incisor, the dentin layer (in blue) had a thickness similar to that of controls, whereas the red/brown enamel layer was hardly visible. **h** The shortened SAB and significant thinned enamel layer underneath the dentin layer (**d**) in the *Satb1*^*−/−*^ mouse incisor are further shown in the enlarged image. Scale bars in **f** and **h**, 50 μM

*Satb1*^*−/−*^ pups generally die around 2.5 weeks after birth [27]. In normal mice, the molars typically have not yet erupted up to this age. Therefore, we focused on the incisor enamel of P13 days mice. In comparison with the age-matched control incisal tip (see Fig. 3a), the erupted portion of the P13 *Satb1*^*−/−*^ incisor was smaller in diameter and more transparent in color (see Fig. 3b). In X-ray radiographic images, well-contrasted enamel layers were evident in the molars and incisors of P13 *wt* mice (indicated by red arrows in Fig. 3c), whereas the enamel layer in *Satb1*^−/−^ mice was significantly thinner and less contrasted compared to the underlying dentin and surrounding alveolar bone (see Fig. 3d). Additionally, comparing the well-developed root furcation beneath the first molar (M1) of wild-type control (indicated by the red dashed line in Fig. 3c), no root furcation could be identified in the age-matched *Satb1*^−/−^ mouse first molar (M1) (see Fig. 3d).

Trichrome staining of the sagittal sections of *wt* P13 incisors showed the mineralizing dentin layer (D) stained blue and the organic enamel layer (E) stained red (see Fig. 3e, f). In the age-matched *Satb1*^*−/−*^ mice, the blue-stained dentin layer (D) had a thickness similar to *wt* controls, whereas the enamel layer (between D and SAB) was significantly thinner (see Fig. 3g, h) as compared to controls.

### Ameloblast morphodifferentiation is compromised in the *Satb1*^*−/−*^ mice

**Fig. 4:**
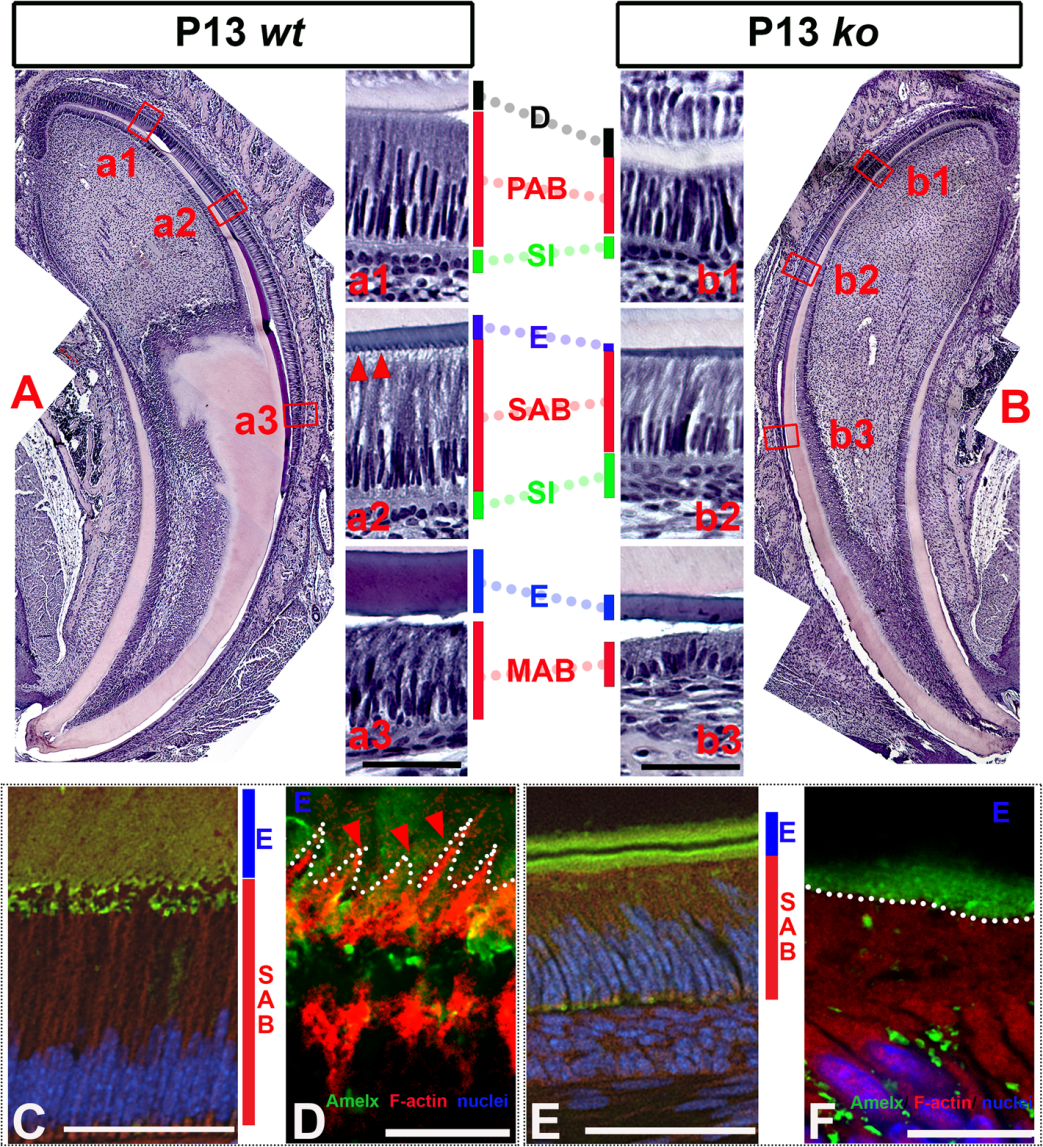
Ameloblast morphology is compromised in the *Satb1*^*−/−*^ mouse incisors. **a** H&E staining of the sagittal sections of the P13 *wt* mouse incisor shows well-developed PABs adjacent to the mineralizing dentin (in pink); SABs and MABs situate next to the enamel layer (in dark red). (a1) PABs, residing between the dentin matrix (**d**) and a layer of stratum intermedium (SI), start to elongate and polarize with the nuclei shifting to basal end (closer to SI). (a2) SABs further elongate and polarize to develop ample supranuclear space to accommodate the increased organelles. Tomes’ processes extending into the enamel layer are present at the apical border of SABs, as indicated by red triangles. (a3) H&E staining shows the reduced MAB situated next to mineralizing enamel matrix (**e**) of *wt* mouse. **b** Ameloblasts at all stages are shorter, and the enamel layer is thinner in the *Satb1*^*−/−*^ mouse incisor as compared to control. (b1) H&E staining shows the shortened PAB in the *Satb1*^*−/−*^ mouse incisor. (b2) SAB in the *Satb1*^*−/−*^ mouse incisor was shortened and lacked Tomes’ processes. (b3) Shortened maturation ameloblasts and a thin enamel layer were detected in the *Satb1*^*−/−*^ mouse incisor. Scale bar in (a3) applied to (a1) and (a2), and in (b3) applied to (b1) and (b2), 50 μM. The picket fence appearance Tomes’ processes at the apical surface of *wt* SABs (see **c**) were visualized by staining the F-actin filament within the cellular protrusion by red fluorescent probe phalloidin, as indicated by red triangles and outlined by the white dotted line (see **d**). Immunostaining with an anti-amelogenin antibody (in green) shows that Tomes’ processes extended and were embedded into the organic enamel matrix (**e**). At the apical surface of *Satb1*^*−/−*^ SABs (see **e**), no organized cellular protrusion could be found to extend into the adjacent *Satb1*^*−/−*^ enamel matrix (**e**, labeled in green). The interface between *Satb1*^*−/−*^ SAB and **e** was outlined by a white dotted line (see **f**). Scale bar in **c** and **e**, 50 μM; in panel **d** and **f**, 10 μM

Standard H&E staining of the P13 sagittal sections of the *Satb1*^*−/−*^ mouse incisors (see Fig. 4b) showed that compared to controls (see Fig. 4a), the ameloblasts in *Satb1*^*−/−*^ mice at all differentiation stages were shorter (see Fig. 4 (b1, b2, b3)). Consistent with those X-ray and trichrome staining results, H&E staining showed a thinner enamel matrix layer (E) in *Satb1*^−/−^ mouse (see Fig. 4 (b2, b3)) as compared to *wt* controls (see Fig. 4 (a2, a3)), though the thickness of the dentin layer was not obviously different. With the use of a Nikon W1-SoRA spinning disk super-resolution confocal microscope, at the apical surface of elongated wild-type SABs (see Fig. 4c), picket fence appearance Tomes’ processes were filled with red phalloidin-labeled F-actin (see red triangles in Fig. 4d), but they were lost at the apical surface of *Satb1*^*−/−*^ ameloblasts (outlined by white dotted line) (see Fig. 4f).

### Amelogenin secretion is impaired in the *Satb1*^*−/−*^ mice

**Fig. 5:**
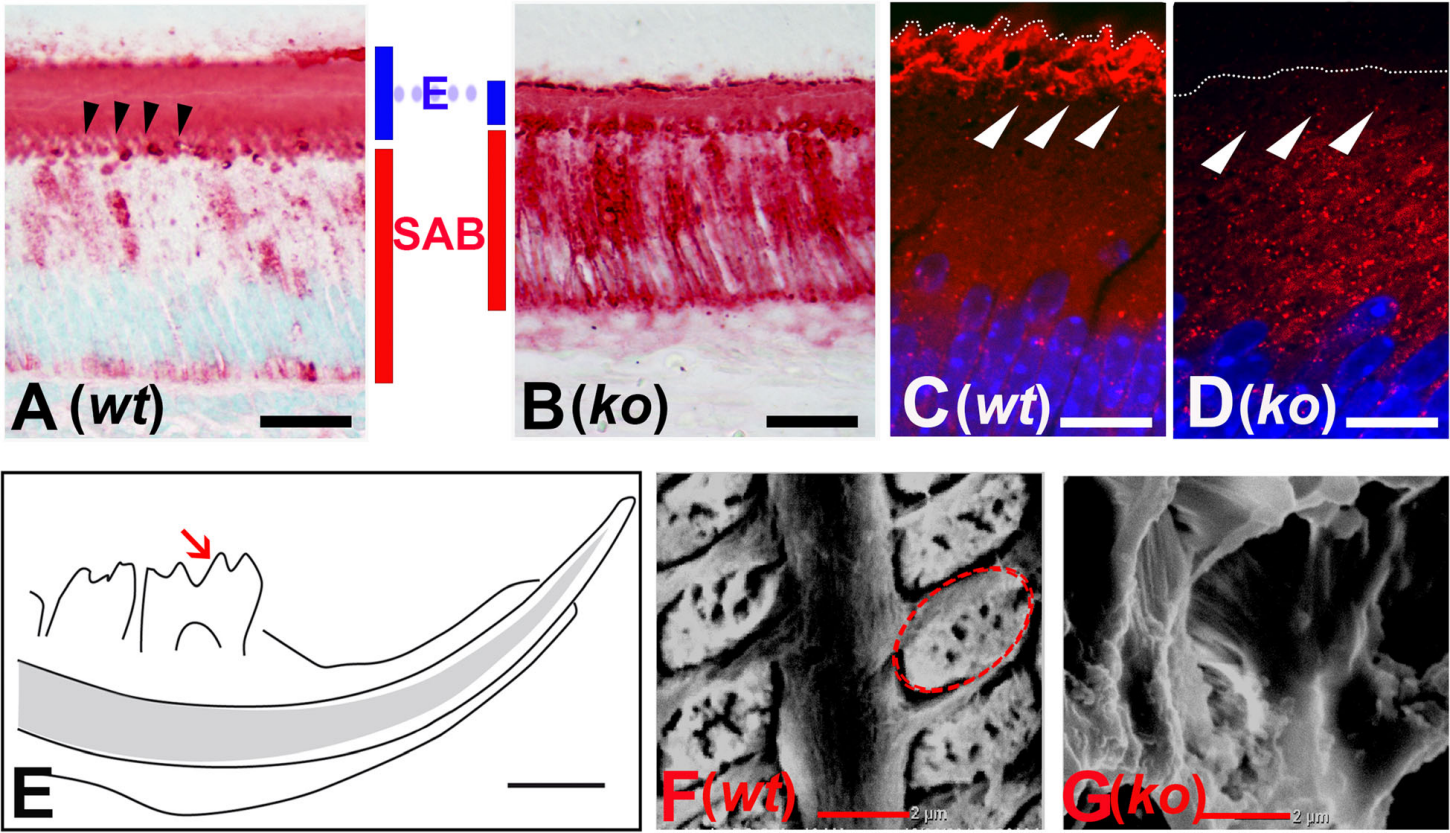
Deficiency of amelogenin secretion results in a thin and disorganized enamel layer in *Satb1*^*−/−*^ mouse. **a** Amelogenin immunostaining on a sagittal section of the P13 *wt* mouse incisor shows amelogenins are primarily detected in the enamel matrix and Tomes’ processes (indicated by black triangles). **b** A representative sagittal section from the P13 *Satb1*^*−/−*^ mouse incisor shows intense amelogenin immunoreactive signal in the shortened ameloblasts, no obvious Tomes’ processes, and a thin layer enamel matrix with a comparable intense immunoreactive signal as that of the control enamel (**e**). Scale bar in **a** and **b**, 25 μM. Rab30 was immunolocalized in the cytoplasm closed to the apical surface of wild-type secretory ameloblasts (as indicated by white triangles) (**c**). More disperse and intense Rab30 immunostaining signal was detected in the cytoplasm, but not at the apical surface (indicated by white triangles) of *Satb1*^*−/−*^ mouse secretory ameloblasts (**d**). The interface between ameloblasts and the enamel matrix was outlined by a white dotted line. Scale bar in panel **c** and **d**, 10 μM. Enamel structure in P13 *Satb1*^*−/−*^ mouse teeth is disorganized. **e** Ground sagittal sections of mouse hemimandible were examined under scanning electron microscope at the position highlighted with a red arrow. **f** Ordered, well-aligned enamel rods were detected in full width of the *wt* incisor enamel layer. The red dotted line traces one intact enamel rod, which is fabricated by one ameloblast. **g** No well-defined structure was visualized in the thin layer of enamel of the *Satb1*^*−/−*^ mouse at the corresponding position. Scale bar in **e**, 1 mm; **f** and **g**, 2 μm

Amelogenins comprise over 90% of the enamel matrix proteins that are synthesized and secreted by ameloblasts during enamel formation. Immunostaining showed that while amelogenins were primarily detected in the enamel matrix (E) residing on SABs in the *wt* mice (see Fig. 5a), greatly increased intracellular amelogenins were detected in the cytoplasm of *Satb1*^*−/−*^ SABs (see Fig. 5b). There was no significant difference in the transcript levels of *Amelx*, encoding amelogenins, in SABs microdissected from the *wt* and *Satb1*^*−/−*^ mouse molars based on semi-quantitative PCR analysis and Student’s *t* test (*n* = 6, *P* > 0.05).

We next addressed if the transport of secretory vesicles would be affected in the ameloblasts of the *Satb1*^*−/−*^ mice. We focused on Rab30 since it is a key component of the transport vesicles facilitating the fusion of vesicles with a targeting membrane [48]. Our microarray analysis showed that Rab30 was increased in secretory ameloblasts up to 4.9-, 2.5-, and 1.9-fold compared to ES-EC, OE, and PAB respectively. Rab30 immunopositive signal was sparsely distributed in the cytoplasm and was more concentrated close to the apical surface of *wt* SABs (see Fig. 5c, indicated by white triangles). By contrast, increased numbers of Rab30-containing puncta were detected in the cytoplasm of *Satb1*^*−/−*^ SABs, consistent with the increased intracellular amelogenin immunostaining (see Fig. 5d). Semi-quantitative PCR analyses demonstrated that the expression levels of *Rab30* gene were similar in *Satb1*^*−/−*^ SABs and *wt* SABs (*n* = 6, *P* > 0.05 by Student’s *t* test). These data indicate that secretory vesicles carrying amelogenins and likely other EMPs cannot be properly transported to the apical plasma membrane in the *Satb1*^*−/−*^ SABs for secretion.

### Abnormal enamel structure is developed on the *Satb1*^*−/−*^ mouse teeth

Scanning electron microscope (SEM) images showed that enamel rods with uniform width and architecture were orderly arranged on the P13 *wt* mouse first molar (see Fig. 5f). No defined enamel rods were observed in the P13 *Satb1*^*−/−*^ mouse first molar enamel at a comparable site as controls (see Fig. 5g). This finding suggests a lack of directional secretion of EMPs into the enamel space.

### Tight junction complex is altered in the *Satb1*^*−/−*^ mouse ameloblasts

**Fig. 6:**
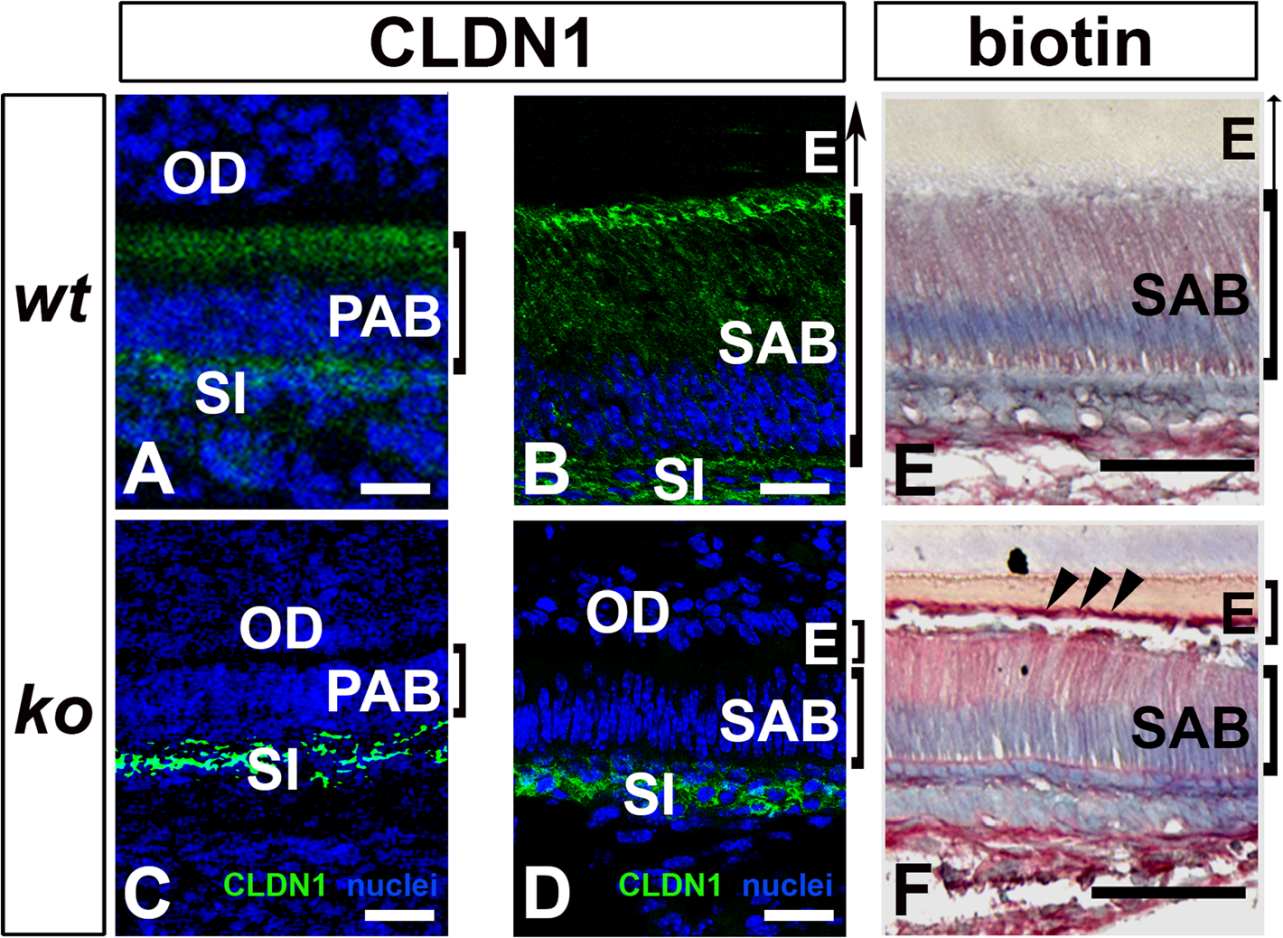
Ameloblast layer’s barrier function is disrupted in *Satb1*^*−/−*^ mouse ameloblasts. **a** Claudin 1 (CLDN1) immunoreactive signal (in green) was detected primarily at the apical end of P1 *wt* presecretory ameloblasts (PAB) and underlying stratum intermedium (SI) **b** and P1 *wt* secretory ameloblasts (SAB) and SI. The cell nuclei were counterstained in blue. **c** CLDN1 immunoreactive signal in *Satb1*^*−/−*^ mouse PAB was primarily localized in SI beneath PAB. **d** Identical immunostaining pattern was found in SI under the *Satb1*^*−/−*^ SAB. OD, odontoblast; E, enamel. Scale bars, 20 μM. **e** A small amount of injected tracer molecule Sulfo-NHS-Biotin was detected in the enamel matrix (**e**) adjacent to *wt* SAB. **f** Significant more tracer molecule Sulfo-NHS-Biotin was found in the enamel matrix (**e**) adjacent to *Satb1*^*−/−*^ mouse SAB and at the surface of the enamel matrix layer. Scale bars: 50 μM

Claudin 1 was immunolocalized primarily at the apical surfaces of presecretory ameloblasts (PAB) and secretory ameloblasts (SAB) and at the membrane of the underlying stratum intermedium (SI) in *wt* mice (see Fig. 6a, b). In the *Satb1*^*−/−*^ mouse incisors, claudin 1 was hardly detected at the apical surface of PAB and SAB (see Fig. 6c, d), though the claudin 1 immunoreactive signal in the stratum intermedium (SI) remained similar to that of control SI (Fig. 6c, d). Impaired claudin 1 distribution indicated the loss of cell polarity in the *Satb1*^*−/−*^ ameloblasts.

Tight junctions are critical for epithelial layer integrity and transcellular barrier function. When tracer molecule Sulfo-NHS-Biotin was injected in mice, only a minimal amount of Sulfo-NHS-Biotin was detected in the enamel matrix (E) adjacent to *wt* SABs (see Fig. 6e). Relatively more Sulfo-NHS-Biotin was found in the enamel matrix (E) adjacent to *Satb1*^*−/−*^ SABs, in particular, at the surface of the enamel matrix layer as pointed by the black arrows (see Fig. 6f), as compared to *wt* controls. These results demonstrated that tight junctions at the apical surface of SABs were impaired by the deletion of SATB1.

### Actin filament assembly at the apical end of SABs is disrupted in the *Satb1*^*−/−*^ mice

**Fig. 7:**
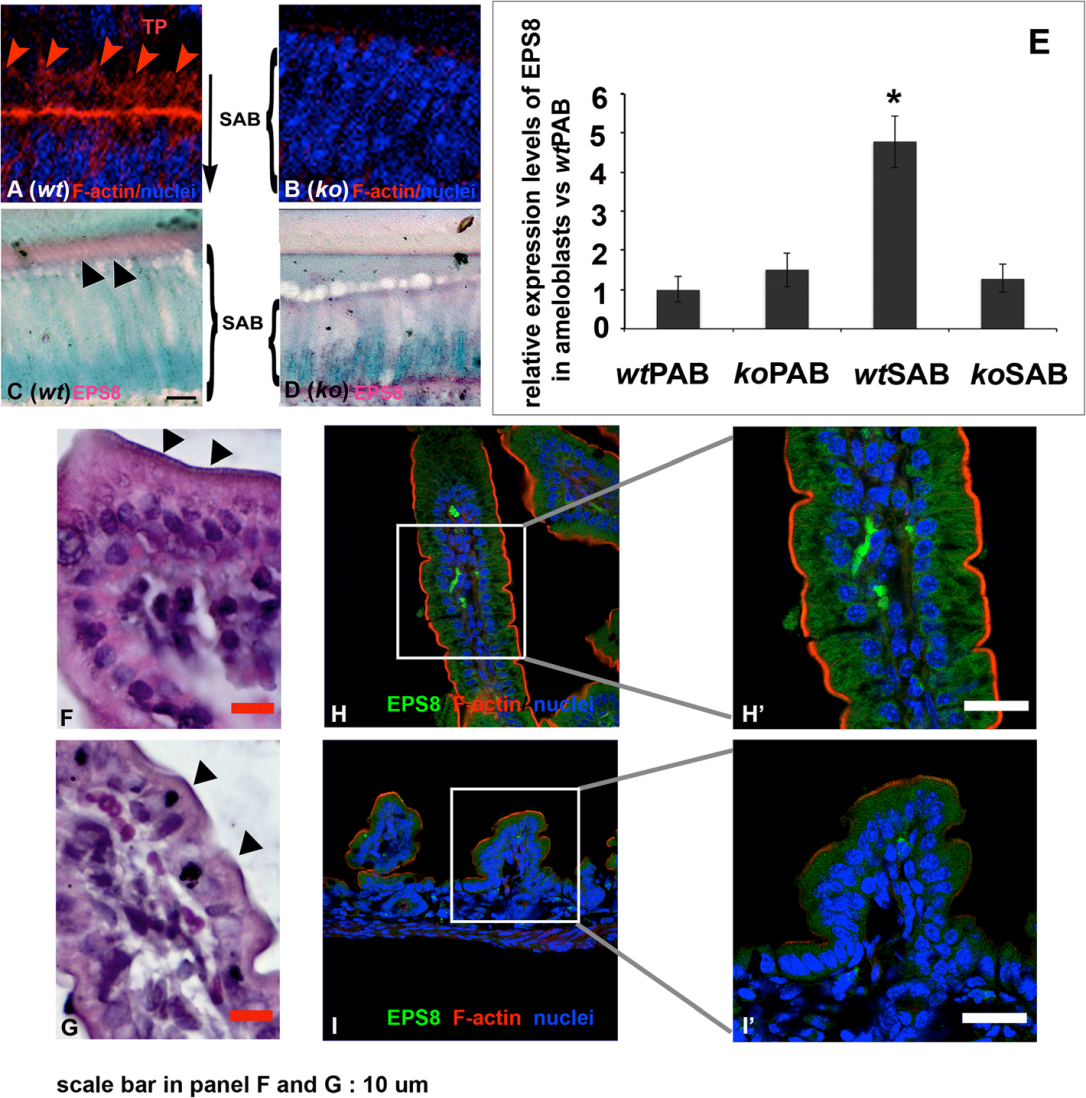
The cytoplasmic protrusion of secretory ameloblast and small intestine epithelial cells are deformed in the *Satb1*^*−/−*^ mice. **A** Circumferential F-actin belt-like structure (in red) was detected along the apical end of *wt* SAB, and branched F-actin bundle was detected in the Tomes’ processes (TP). **B** No apparent F-actin assembly was detected at the apical end of the *Satb1*^*−/−*^ SAB. **C** EPS8 was immunolocalized in the Tomes’ processes (indicated by black triangles) of *wt* SAB. **D** There was no Tomes’ process and concentrated EPS8 signal that could be detected in the *Satb1*^*−/−*^ SAB. **E** qPCR analyses on ameloblasts collected from mouse first molars showed that EPS8 was significantly upregulated up to 5.6-fold in *wt* SAB vs *wt* PAB. However, EPS8 expression levels in the *Satb1*^*−/−*^ PAB and SAB were similar to that detected in *wt* PAB. **F** The well-developed microvilli (indicated by black triangles) were detected along the lining of *wt* mouse small intestine. **G** Microvillus layer on the surface of the *Satb1*^*−/−*^ mouse small intestine was thinner and less consistent (indicated by black triangles). **H**, **H′** Immunofluorescent staining with EPS8 antibody demonstrated that EPS8 was concentrated and aligned toward wild-type mouse small intestine brush border (in green). Phalloidin-labeled F-actin was well distributed along the brush border (in red). **I**, **I′** EPS8 immunoreactive signal (in green) and F-actin architecture (in red) were less intense and organized at the apical surface of *Satb1*^*−/−*^ mouse intestinal epithelial cells. Scale bars in **F**, **G**, **H′**, and **I′**, 10 μM

Claudins are responsible for assembling perijunctional tensile ring/belt of actin filaments at the apical end of polarized columnar epithelial cells [49]. Phalloidin staining showed that the condensed perijunctional tensile ring/belt-like actin filaments were stained in red at the apical end of control SABs (see Fig. 7A). Consistent with the reduced claudin 1 immunostaining, a condensed and orderly actin filament ring/belt-like structure could not be detected at the apical end of *Satb1*^*−/−*^ mouse SABs (see Fig. 7B). Interestingly, the branched F-actin bundle was stained in red inside of Tomes’ processes (TP) in the *wt* mice (see Fig. 7A), which could not be detected at the apical end of *Satb1*^*−/−*^ mouse SABs. Epidermal growth factor receptor pathway substrate 8 (EPS8), previously linked to small intestinal epithelial cytoplasmic protrusion formation [50–53], was mostly immunolocalized in the Tomes’ processes at the apical surface of *wt* SABs (see Fig. 7C), but not at the same location of *Satb1*^*−/−*^ mouse SABs (see Fig. 7D). qPCR analyses on the PABs and SABs collected from the mouse first molars showed that *EPS8* was significantly upregulated up to 5.6-fold in *wt* SABs vs *wt* PABs (*n* = 6, *P* < 0.05 by one-way ANOVA with post hoc tests). However, EPS8 expression levels in *Satb1*^*−/−*^ mouse PABs and SABs were similar to those detected in *wt* PABs (see Fig. 7E). Thus, these data suggest that SATB1 regulates actin filament assembly and Tomes’ process formation through regulating the expression of *EPS8* in secretory ameloblasts.

In human breast cancer cell line MDA-MB-231, the expression of EPS8 and CLDN1 genes depends on SATB1 [22], and SATB1 binding sites have been found at multiple sites near and within genes encoding CLDN1 and EPS8 (based on our unpublished ChIP-seq results). To further investigate the regulatory function of SATB1 on the expression of CLDN1 and EPS8, we manipulated the expression levels of *SATB1* in human ameloblast-lineage cells (ALCs) using Retro-X™ Tet-On Advanced Inducible Expression System. When *SATB1* expression levels increased about 2.5-fold compared to that of non-transduced ALCs, *CLDN1* and *EPS8* expression levels were about 4.3-fold and 0.43-fold as compared to that of controls, respectively. However, when SATB1 expression levels increased 38-fold, claudin 1 significantly reduced to 0.27-fold, but EPS8 increased to 5-fold as compared to that of non-transduced ALCs (*n* = 6, *P* < 0.05). In short, repression of claudin 1 and enhancement of EPS8 by SATB1 could be recapitulated in ALCs by SATB1 overexpression.

Microvilli, constituting the brush border of the small intestine, are an array of actin filament-support apical membrane protrusion of enterocytes [54]. The identical thickness of microvilli (indicated by black arrows in Fig. 7F) was detected along the lining of *wt* mouse small intestine. Microvillus layer on the surface of *Satb1*^*−/−*^ mouse small intestine was thinner and less consistent (see Fig. 7G). In addition, small intestine epithelial cells in *Satb1*^*−/−*^ mouse were shorter, and the nuclei were less polarized. Immunofluorescent staining demonstrated that EPS8 was concentrated at the site of *wt* mouse small intestine brush border (see Fig. 7H, H′), but was less intense at the apical surface of *ko* mouse small intestine (see Fig. 7I, I′). There was a threefold reduction in the EPS8 expression levels in the *Satb1*^*−/−*^ mouse small intestine as compared to that of controls by qPCR (*n* = 6, *P* < 0.05 by Student’s *t* test). The defective EPS8 expression and microvillus formation in *Satb1*^*−/−*^ mouse small intestine epithelial cells further demonstrated the important roles of SATB1 on actin filament assembly and cytoplasmic protrusion formation. Actin filaments underneath the plasma membrane function as cables for short-distance transport, moving the vesicles to fuse with the target membrane [1].

## Discussion

Epithelial cell polarity is a feature of asymmetric sequestration of polarity proteins and macromolecules into specific cellular domains. Establishment and maintenance of epithelial cell polarity are critical for many cellular functions, such as cell division, polarized transport, cellular protrusion, and migration [55]. Our studies showed that SATB1 was spatially and temporally upregulated in ameloblast lineage during tooth development. A loss-of-function *Satb1*^*−/−*^ mouse model demonstrated that SATB1 is indispensable for ameloblast morphogenesis and normal enamel formation.

Ameloblasts undergo remarkable morphological modulation as they differentiate from cuboidal dental epithelial precursor cells to polarized tall columnar secretory ameloblasts that secrete enamel matrix proteins, composed of 90% amelogenins, to set out the construction of the enamel. Amelogenins are necessary for the hydroxyapatite deposition, growth, and full thickness of enamel formation [56]. Ameloblasts in *Satb1*^*−/−*^ mice were shorter in length and lack polarity as compared to *wt* ameloblasts at all stages. The fact that the *Satb1* null mice often die prematurely constrained us to focus on PAB and SAB.

The increased amount of amelogenins in the *Satb1*^*−/−*^ mouse SABs, while amelogenin mRNA transcript levels in these cells remained unchanged as compared to that of the controls, indicates defects in amelogenin secretion into the extracellular enamel space. Increased Rab30 dispersed throughout the cytoplasm of *Satb1*^*−/−*^ SABs provided additional evidence to demonstrate the vesicle accumulation in these cells. Rab30 was the major member of Rab GTPase family expressed by *wt* SABs according to the microarray analysis and was primarily localized in the cytoplasm proximal to the nuclei of *wt* SABs. Rab30 is a key coordinator of eukaryotic intracellular membrane trafficking, primarily associated with vesicle budding and formation along the secretory pathway [48]. A massive accumulation of amelogenins and Rab30 highly accounts for the thin, hypomineralized, and non-prismatic enamel layers on both incisors and molars of the *Satb1*^*−/−*^ mice.

Tomes’ processes (TP) are the landmark structures at the apical surface of *wt* SABs, through which the secretory vesicles fuse with the distal faces of TPs to directionally release EMPs into the enamel matrix [57]. In the *Satb1*^*−/−*^ mice, secretory ameloblasts lost their apical Tomes’ processes. As secretory ameloblasts lose polarity and Tomes’ processes, secretory vesicles likely fail to be unidirectionally transported and fused with the plasma membrane. This would impair the directional release of amelogenins and other enamel matrix proteins into the enamel space, resulting in defective enamel. Cytoskeleton organization plays important roles in delivering the vesicles and cargos to the correct domains [58]. Filamentous actin meshwork branches out in Tomes’ processes of *wt* secretory ameloblasts and are thought to serve as tracks to transport secretory vesicles through the cytoplasm to the secretory face of ameloblast Tomes’ processes [59, 60]. Our data indicated that SATB1 deficiency affected filamentous actin assembly and arrangement underneath the apical plasma membrane of SABs, which might impede the routes for secretory vesicle transporting to secretory front.

There is scant information regarding the molecular mechanisms contributing to the formation of Tomes’ processes. In the intestinal epithelial cells, elongation of F-actin meshwork proximal to the plasma membrane is believed to generate the pushing force for cell protrusion and microvillus formation. Similarly, build-up of an F-actin meshwork might also direct the formation of ameloblast Tomes’ processes. Based on the knowledge learned from intestinal epithelial cells, we know signaling activated by cell-ECM adhesion recruits the formation of focal adhesion complex, which activates the assembly of F-actin branches to anchor cells and generates pushing forces for modulating the cell cortex structure and movement [61, 62]. Similar to the lack of Tomes’ processes in secretory ameloblasts, microvilli in the *Satb1*^*−/−*^ mouse small intestinal epithelial cells were deformed as well. We further demonstrated that EPS8 was downregulated in both small intestinal epithelial cells and secretory ameloblasts from *Satb1*^*−/−*^ mice. EPS8 is necessary for the formation of small intestinal microvilli, which binds filament actin together to stabilize actin bundling in intestinal epithelial cells [52, 63–65]. We ascertain that EPS8 is one of the SATB1-influenced genes that may be responsible for the cytoplasmic protrusion defect in the *Satb1*^*−/−*^ mice. We also found by qPCR analysis that fromin1, a promoter for actin filament nucleation and polymerization [66, 67], was also downregulated in both *Satb1*^*−/−*^ mouse secretory ameloblasts and small intestinal epithelial cells by qPCR. We have not been able to confirm the altered distribution pattern of formin1 in the *Satb1*^*−/−*^ secretory ameloblasts due to the lack of an antibody. We assume that SATB1 can regulate secretion through regulating the expression of genes associated with cytoskeletal filamentous actin organization.

Another mechanism that may also contribute to the defective enamel in *Satb1*^*−/−*^ mice is the increased paracellular permeability between ameloblasts, resulting from the significantly reduced and disorientated claudin 1 distribution at the apical end of ameloblasts when the *Satb1* gene is not present. Intercellular tight junctions assemble at the apical end of ameloblasts to physically connect single layer of tall columnar ameloblasts together into a sturdy sheet and control the permeability of molecules and ions through the paracellular space. These structural components are essential for the maintenance of the unique enamel space microenvironment. The increased trans-ameloblastic permeability could potentially affect the ion concentration, osmotic pressure, and pH value in the enamel matrix fluid, in turn, to collectively deteriorate all aspects of enamel formation, including ameloblast-matrix adhesion, cell shaping, enamel matrix protein secretion, and EMP enzymatic processing, which would in turn alter the enamel crystal growth and mineralization.

Secretory ameloblasts have very unique cellular morphology and functions as compared to other epithelial cells, even ameloblasts at other stages. Thus, SAB morphology and functions must be achieved by the coordination of many genes. Through our transcriptome microarray analyses, the second most upregulated transcriptional regulator in SAB is c-Maf. We initially studied the potential link between transcription factor c-Maf and SATB1 and found that c-Maf requires SATB1 for its expression in ameloblasts (see Additional file 3: Figure S3). This is similar to the scenario found during T helper 2 activation, where SATB1 induces c-Maf expression and recruits c-Maf to the promoter region of specific cytokine genes [21]. During ameloblast differentiation, c-Maf as well as other transcriptional regulators, for instance, RNA polymerase II elongation factor (such as ELL2), may be guided by a SATB1 network to be recruited and assembled at specific gene loci to achieve a large-scale gene regulation, enabling ameloblast differentiation.

**Fig. 8:**
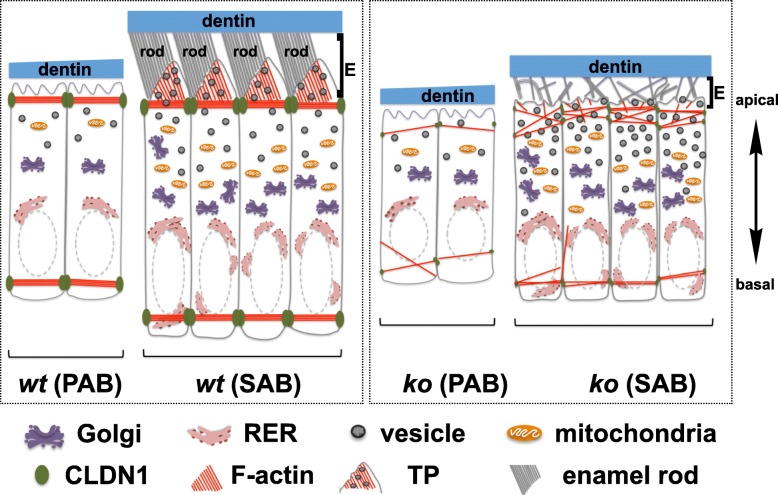
Model of effects of *Satb1* deletion on enamel formation. During tooth development, the expression of SATB1 starts in PABs, which attach to the dentin matrix. PABs further elongate and polarize as they differentiate into SABs. Circumferential F-actin belt-like structures are assembled along apical poles, mostly attributing to the locally increased claudin 1 (CLDN1). These F-actin belt-like structures not only physically connect elongated ameloblasts into a sturdy sheet, but also control the contents to be secreted as a sieve. As SABs start to secrete enamel matrix, cells move away from the matrix leaving a cytoplasmic protrusion surrounding by the organic matrix. This protrusion is a histological landmark of SAB, so-called Tomes’ process (TP). The F-actin networks built along the cytoplasmic membrane of the apical surface, likely through the interactions between transmembrane integrins and extracellular matrix proteins, could generate pushing force to facilitate the membrane protrusion and serve as tracks for transporting vesicle/A (secretory vesicles containing amelogenins). TP is responsible for the directional secretion of enamel matrix proteins and the formation of one enamel rod. When *Satb1* gene is deleted, PABs are significantly shorter and have barely detectable CLDN1 at the apical pole. Not surprisingly, no F-actin belt orderly assembles at the apical pole of PABs. SABs are continuously shorter compared to controls with significantly reduced CLDN1, deformed F-actin architecture, and no Tomes’ process at its apical end. These defects are believed to account for the amelogenin retention in *Satb1*^*−/−*^ ameloblasts and a thin, hypomineralized and disordered enamel phenotype. It is worthy to note that often several claudin family members are co-expressed and interact with each other to regulate the tight junction structure in epithelial cells. This is a simplified schema that primarily focuses on the correlation between SATB1 with CLDN1

Taken together, our data shows that chromatin organizer SATB1 is temporarily upregulated in epithelially derived ameloblasts and appears to affect ameloblasts’ tight junction formation, polarity, actin filament assembly, Tomes’ process formation, and polarized secretion. All of these events are critical to ameloblast morphodifferentiation and normal enamel formation (illustrated in Fig. 8). These findings suggest the potential application of SATB1, perhaps in concert with other key transcriptional regulators, for enamel regeneration by regulating ameloblast programming from either oral epithelial cells or stem cell-derived epithelial cells.

## Material and methods

### To characterize the gene expression profile of stage-specific ameloblasts by whole-transcript microarray analyses

#### Preparation of human embryonic stem cells into epithelial cells

Human embryonic stem cells (hESC) (NIH-registered WA09 cell line) were purchased from the WiCell Institute (WI, USA). These stem cells were maintained as previously reported, and their differentiation into epithelial cells (ES-ECs) was induced and characterized according to the established protocols [68, 69]. These cells served as controls for microarray analyses.

#### Laser microdissection of human fetal oral buccal mucosal epithelial cells and ameloblasts

Tissues were collected from 18- to 19-week human aborted fetal cadaver tissues, under guidelines approved by the Committee on Human Research at UCSF, with patients’ consent. Fetal buccal mucosa and maxillae were isolated and embedded in O.C.T. (Tissue-Tek® optimum cutting temperature compound). Those tissues were then cryosectioned at a thickness of 10 μm per section, and consecutive sections were collected on Leica PEN-membrane slides. After stained with hematoxylin & eosin, specific cell types were harvested using a P.A.L.M Laser Dissecting Microscope (Zeiss). Oral buccal mucosal epithelial cells (OE) were collected from the cheek tissues. Presecretory ameloblasts (PABs) were identified as the single layer of columnar epithelial cells directly bordering with the dentin matrix. Secretory ameloblasts (SABs) were identified as the elongated and polarized epithelial cells adjoining to the organic enamel matrix (shown in Fig. 1a). PABs or SABs from three separate maxillae were pooled into one collection tube as a single sample. Three samples were collected for each of four groups, including ES-EC, OE, PAB, and SAB. The microdissected cells were preserved in lysis buffer (RLT buffer) provided by QIAGEN RNeasy Micro kit, 1% β-mercaptoethanol (Sigma-Aldrich) prior to RNA purification.

#### Affymetrix whole-transcript human gene 1.0ST microarray

Total RNA was purified from ES-EC, OE, PAB, and SAB cells using RNeasy Micro Kit (QIAGEN, Valencia, CA). The RNA concentration was determined using a NanoDrop ND-1000 (Thermo Scientific), and only the RNA samples with A_260_:A_280_ ratio between 7 and 9, determined by a 2100 BioAnalyzer (Agilent Technologies), were subjected to microarray analyses. Fifty nanograms total RNA from each sample was used for RNA amplification and antisense cDNA synthesis with Ovation Pico WTA System (NuGEN Technologies). The biotinylated ST-cDNA probes were generated as previously mentioned [70] and hybridized onto a GeneChip® of Human Gene 1.0 ST Array (Affymetrix, Santa Clara, CA) for 16 h at 45 °C. After hybridization, the array chips were stained with streptavidin phycoerythrin and imaged using an Affymetrix Gene Chip Scanner 3000 (Affymetrix, Santa Clara, CA).

#### Significantly differentially expressed gene profile analyses

The robust multichip average (RMA) method was used for background adjustment, quantile normalization, and median polish summarization using Affymetrix Expression Console Software. These normalized and summarized raw data were submitted to the Gene Expression Omnibus database (GEO accession number is GSE75954). RMA data were analyzed using Partex Genomics Suite software (St. Louis, MI) to generate a principal component analysis (PCA) clustering plot. Both R software environment with the Bioconductor affy package [71, 72] and GeneSifter software (Geospiza, Inc., WA) were used to generate the lists of significantly differentially expressed genes in PAB and SAB as compared to non-dental epithelial cells (average expression levels of ES-EC plus OE), using a cutoff of ± 2.0-fold change and *p* < 0.05. These lists were further analyzed using Exploratory Gene Association Networks (EGAN) software [73] to identify and summarize enriched GO functional pathways; the results of which are reported in Fig. 1b.

#### Validation of expression levels of genes of interest by semi-quantitative PCR

Total RNA purified from ES-EC, OE, PAB, and SAB cells was used to synthesize cDNA libraries with SuperScript™ III First-Strand Synthesis System (Life Technologies). Semi-quantitative PCR was performed using the ABI 7500 system (Applied Biosystems). Primers and corresponding TaqMan® probes used to amplify endogenous control *GAPDH* and all target genes were purchased from Applied Biosystems. To determine the relative expression level of the target gene, the comparative CT (threshold cycle) method was used [74].

### Characterization of enamel mineralization by X-ray analysis

The generation of *Satb1*^*−/−*^ mouse model was previously reported by Dr. Kohwi-Shigematsu et al. [27]. The animals were maintained in the UCSF animal care facility, which is a barrier facility, accredited by the Association for Assessment and Accreditation of Laboratory Animal Care (AAALAC). Hemimandibles from P13 *wt* and *Satb1*^*−/−*^ mice were radiographed using a Faxitron X-ray system (Hewlett-Packard, Model 43855A) at 30 kV for 2 min at a focal distance of 46 cm with the microfine grain emulsion films (type 4489, Kodak, Rochester, New York).

### Characterization of enamel rods by scanning electron microscopy

To characterize the size and arrangement of the enamel rods in *wt* and *Satb1*^*−/−*^ mice using scanning electron microscope, three sets of hemimandibles from P13 *wt* and *Satb1*^*−/−*^ mice were dissected, embedded in epoxy, and polished in a sagittal orientation using a series of SiC paper and diamond polishing suspension. Subsequently, specimens were etched with 10 mM HCl for 60 s. Specimens were sputter-coated with Au–Pd, and enamel layers of the first molar were imaged at 10 kV using a NeoScope SEM (JEOL) as described previously [75].

### Characterization of the histomorphometry of the enamel matrix and ameloblasts

We used the continuously growing mouse incisors, including all developmental stages of ameloblasts on a single entity, to characterize ameloblast/enamel phenotype from *wt* and *Satb1*^*−/−*^ mice. We determined the distribution of SATB1 and proteins associated with epithelial apical polarity (CLDN1:claudin 1), actin filament assembly (EPS8), and vesicle membrane fusion (Rab30). Adult C57BL/6J mice were anesthetized with 240 mg/kg tribromoethanol (Sigma-Aldrich, St. Louis, MO), and perfuse-fixed with 4% paraformaldehyde (PFA). The hemimandibles were dissected and post-fixed with 4% PFA for 24 h at 4 °C followed by decalcification in 8% EDTA at 4 °C for 4 weeks. The hemimandibles were then processed, embedded, and sectioned sagittally. The sections were stained with either H&E or Goldman’s trichrome for ameloblast and enamel matrix phenotypical analyses [76].

For immunostaining, the sagittal sections were boiled in 10 mM citrate buffer (pH 6.0) for 20 min to retrieve antigens. Next, the sections were incubated with 10% swine and 5% goat sera followed by incubation with primary antibodies recognized antigens SATB1 (abcam®), CLDN1 (abcam®), EPS8 (Santa Cruz Biotechnology), Rab30 (Novus Biologicals), or amelogenins [77] overnight at 4 °C. The sections were then incubated with biotin- or fluorescein-conjugated species-specific secondary antibody for 1 h at RT. To detect the biotin-labeled antibody, the sections were next incubated with alkaline phosphatase-conjugated streptavidin (Vector Laboratories, Inc.) for 30 min then incubated with Vector® Red kit (Vector Laboratories, Inc.) to reveal positive reactivity indicated by the presence of pink/red precipitating reaction product. Counter-staining was performed with methyl green (Dako). The sections incubated with fluorescein were then counterstained with 1 μg/ml Hoechst (Life Technologies).

To label cytoskeleton filamentous actin, the hemimandibles collected from P1 *wt* and *Satb1*^*−/−*^ mice were fixed in 4% formaldehyde/cytoskeleton-stabilizing BRB80 buffer (80 mM PIPES; 1 mM EGTA, and 1 mM MgCl2, pH 6.8) at 37 °C for 2 h then embedded in the OCT compound. Incisor sagittal cryosections with a thickness of 5 μm for each were collected. After blocking with 3% BSA, the sections were incubated with Alexa Fluor® 594-phalloidin (Life Technologies) for 20 min to label F-actin. After stringent washes, the cell nuclei were counterstained with 1 μg/ml. The slides were imaged using a high-resolution and high-speed Leica TCS SP5 spectrum confocal microscope or a Nikon W1-SoRA spinning disk super-resolution confocal microscope.

### Characterization of the transcellular barrier function of secretory ameloblast layer of *wt* and *Satb1*^*−/−*^ mice

The apical tight junction is an important indicator for ameloblast polarity and can be measured by the functional selectivity of the paracellular apical barrier. Sulfo-NHS-Biotin was used as a tracer molecule to evaluate the effects of SATB1 on the ameloblast layer barrier function as previously described [78]. Briefly, four 10-day old pups from each *wt* and *Satb1*^*−/−*^ mouse model were anesthetized by intraperitoneal injection with 0.05 ml/10 g of ketamine (18 mg/ml). A 30-G injection needle with a blunt end was carefully inserted into the left ventricle of the heart, and 150 μl/g of Sulfo-NHS-Biotin solution was perfused into the bloodstream. Ten minutes after receiving Sulfo-NHS-Biotin injection, the pups were sacrificed, and the hemimandibles were dissected, processed, and paraffin-embedded for histological sectioning as previously mentioned. The sagittal sections were blocked with 5% BSA for 1 h then incubated with alkaline phosphatase-conjugated streptavidin (Vector Laboratories, Inc.) for 30 min, and immunoreactivity was visualized using a Vector® Red kit (Vector Laboratories, Inc.).

### Measurement of the relative amelogenin expression levels in *Satb1*^*−/−*^ mouse ameloblasts

After euthanizing with carbon dioxide asphyxiation followed by cervical dislocation, the mandibles were dissected from P5 control and *Satb1*^*−/−*^ mice. Ameloblasts were removed from the first molars, which have the enamel primarily at the secretory stage [79] were microdissected from the surface of the teeth. The total RNA was then purified, and qPCR was performed to determine the relative amounts of amelogenin mRNA in microdissected controls as compared to *Satb1*^*−/−*^ SABs*.* The primer sequences for the amelogenin gene were as follows: mouse *amelogenin* sense-GGGACCTGGATTTTGTTTGCC, antisense-TTCAAAGGGGTAAGCACCTCA.

### Investigation of the effects of overexpressed SATB1 on primary human ameloblast lineage cells

Human ameloblast lineage cells (hALCs) were harvested from the tooth buds as mentioned above. We followed the protocol previously established in our laboratory to maintain these cells [80–82]. As the first passage of hALCs reached 80% confluence, the KGM2 medium was replaced with a D-MEM medium with 10% FBS and 5 μg/ml Polybrene (Sigma-Aldrich). Following Clontech’s Retro-X™ Tet-On Advanced Inducible Expression System manufacturer instructions, expression vector pRetroX-Tight-Pur-human *Satb1* cDNA and *env* expression vector pVSV-G were co-transfected into packaging GP2-293 cells to produce retrovirus stocks. Next, retrovirus was used to infect the hALCs transduced with RetroX-Tet-On expression system. Double-stable hALCs were selected with 400 μg/ml G418 and 1 μg/ml purimycine. Afterwards, 500 ng/ml doxycycline was supplemented to the culture medium to induce the expression of SATB1. The relative expression levels of SATB1 by cells multiplied from each individual clone were measured by qPCR. Two clones with distinctive expression levels of SATB1 were further analyzed for the relative expression levels of CLDN1 and EPS8. The sequences of the primers were as follows: human *SATB1* sense-TCGGGCCATCTGATGAAAACC, antisense-CCAACCTGGATTAGCCCTTTG; human *CLDN1* sense-CGATGAGGTGCAGAAGATGA, antisense-AAGGCAGAGAGAAGCAGCAG; human *EPS8* sense-TCTTCACCACCCTATTCCCAG, antisense-CATCTTTCCGATCCAGCACGA; human *GAPDH* sense-CATGAGAAGTATGACAACAG, antisense-GTGATGGCATGGACTGTGGT.

## Supplementary information

**Additional file 1: Figure S1.** Principal component analysis (PCA) shows a scatter plot of variables of four target cell types, as analyzed using the Partex Genomic Suite software. In this plot, each sphere represents one sample. Spheres in the same color represent three triplicate samples of each cell type. The distance between any groups of cell type is related to the similarity between the two observations in the biplot. This result is depicted two dimensionally with PC1 (34.4%) and PC2 (13.3%) as the X and Y axes, respectively. This mapping indicated that variables of PABs and SABs scattered more closely to each other than to others meaning their closer biological relevance during development. (PSD 1137 kb)

**Additional file 2: Figure S2.** Quantitative PCR verified that genes associated with ameloblast morphogenesis and enamel matrix mineralization are significantly upregulated in microdissected human ameloblasts, in a similar pattern shown by microarray analysis. Genes associated with ameloblast morphogenesis including CLDN1 and ITGA2, enamel matrix formation including AMELX, AMBN, ENAM, MMP20, AMTN, DSPP, DMP1 and ALP, and two transcriptional regulators SATB1 and c-Maf, not previously connected with amelogenesis, were confirmed to be specifically upregulated in PAB and SAB by qPCR. (PSD 1104 kb)

**Additional file 3: Figure S3.** c-Maf protein is reduced in *Satb1*^*-/-*^ mouse incisor ameloblasts. A) In *wt* P13 mouse incisor, positive c-Maf immunostaining signal was visualized in cells within cervical loop (CL, a1), more intensely in presecretory ameloblasts (PAB, a2), less intensely in secretory ameloblasts (SAB, a3), again increased in transitional stage ameloblasts (TAB, a4) and least intensely in early maturation stage ameloblasts (MAB, a5). B) In P13 *Satb1*^*-/-*^ mouse incisors, c-Maf immunoreactive signal was reduced in all stages of ameloblasts (b1-b5). Immunoreactivity appeared to be unaffected in the stratum intermedium (SI) and papillary layer (PL) (b2-b5) when *Satb1* gene is deleted. Scale bar in a1 applied to a2-5 and b1-b5: 20 μM. (PSD 10438 kb)

## Data Availability

The datasets used and/or analyzed during the current study are available from the corresponding author on reasonable request. The microarray data associated with this study has been submitted to the Gene Expression Omnibus database, accession number GSE75954.
